# Ancestral Origin of the ATTCT Repeat Expansion in Spinocerebellar Ataxia Type 10 (SCA10)

**DOI:** 10.1371/journal.pone.0004553

**Published:** 2009-02-23

**Authors:** Teresa Almeida, Isabel Alonso, Sandra Martins, Eliana Marisa Ramos, Luísa Azevedo, Kinji Ohno, António Amorim, Maria Luiza Saraiva-Pereira, Laura Bannach Jardim, Tohru Matsuura, Jorge Sequeiros, Isabel Silveira

**Affiliations:** 1 UnIGENe, IBMC- Instituto de Biologia Molecular e Celular, Universidade do Porto, Porto, Portugal; 2 IPATIMUP-Instituto de Patologia e Imunologia Molecular da Universidade do Porto, Porto, Portugal; 3 Division of Neurogenetics and Bioinformatics, Center for Neurological Diseases and Cancer, Nagoya University Graduate School of Medicine, Nagoya, Japan; 4 Faculdade de Ciências, Universidade do Porto, Porto, Portugal; 5 Hospital de Clínicas de Porto Alegre, Porto Alegre, Brazil; 6 ICBAS, Universidade do Porto, Portugal; Vrije Universiteit Medical Centre, Netherlands

## Abstract

Spinocerebellar ataxia type 10 (SCA10) is an autosomal dominant neurodegenerative disease characterized by cerebellar ataxia and seizures. The disease is caused by a large ATTCT repeat expansion in the *ATXN10* gene. The first families reported with SCA10 were of Mexican origin, but the disease was soon after described in Brazilian families of mixed Portuguese and Amerindian ancestry. The origin of the SCA10 expansion and a possible founder effect that would account for its geographical distribution have been the source of speculation over the last years. To unravel the mutational origin and spread of the SCA10 expansion, we performed an extensive haplotype study, using closely linked STR markers and intragenic SNPs, in families from Brazil and Mexico. Our results showed (1) a shared disease haplotype for all Brazilian and one of the Mexican families, and (2) closely-related haplotypes for the additional SCA10 Mexican families; (3) little or null genetic distance in small normal alleles of different repeat sizes, from the same SNP lineage, indicating that they are being originated by a single step mechanism; and (4) a shared haplotype for pure and interrupted expanded alleles, pointing to a gene conversion model for its generation. In conclusion, we show evidence for an ancestral common origin for SCA10 in Latin America, which might have arisen in an ancestral Amerindian population and later have been spread into the mixed populations of Mexico and Brazil.

## Introduction

Spinocerebellar ataxia type 10 (SCA10 (MIM 603516)) is an autosomal dominant neurodegenerative disease characterized by cerebellar ataxia and seizures [1]. The first descriptions of SCA10 came from families of Mexican origin [1]–[3], but the disease was later identified in Brazilian families of mixed Portuguese and Amerindian ancestry [4]–[5] and most recently in an Argentinian family of mixed Amerindian and Spanish origin [6]. The disease is caused by a large expansion of a pentanucleotide ATTCT repeat in intron 9 of the *ATXN10* gene [3]. The repeat is polymorphic, with normal alleles ranging from 10 to 29 and expanded alleles having 800 to 4500 pentanucleotide repeats. Reduced penetrance has been found for intermediate size alleles of 280–850 repeats [5], [7]. The reported SCA10 families with Amerindian and Spanish admixture present a cerebellar syndrome and epilepsy, whereas those with Portuguese admixture have a cerebellar phenotype but no seizures in most cases [4]–[5].

The ancestral origin of the SCA10 expansion and a possible founder effect that would explain the American distribution of the disease have been the source of debate over the last years [1], [5]–[8]. In Brazil, the disease is the second most common SCA in the Southern State of Paraná [4], but it is also present in the closer State of Rio Grande do Sul [5] and most probably in other not yet studied regions. In Mexico, this disorder is also one of the most common SCAs [9]. A founder effect could account for the frequency of SCA10 in Latin America; the mutation would have arisen in the Amerindian population and later would have been spread into the mixed populations of Mexico and Brazil. The common Amerindian ethnic origin of the SCA10 families, together with the absence of SCA10 in several European populations, including Spanish and Portuguese families [5], [8], [10], strongly supports a founder effect for SCA10. In an attempt to gain insight into the ancestral origin and spread of the SCA10 mutation, we performed an extensive haplotype study using two closely linked STRs and four intragenic SNPs, in families from Brazil and Mexico. The slowly evolving SNPs and rapidly evolving STRs were used to dissect the origins and evolution of SCA10 affected chromosomes. Information given by the stable SNPs provides evidence on ancestral lineages and their original background, whereas the fast evolving STRs are useful to estimate the antiquity of affected haplotypes. Here we present evidence that supports the existence of one intragenic haplotype associated with the SCA10 expansion in Latin American families, pointing to an ancestral Amerindian origin for this mutation.

## Results

**Figure 1:**
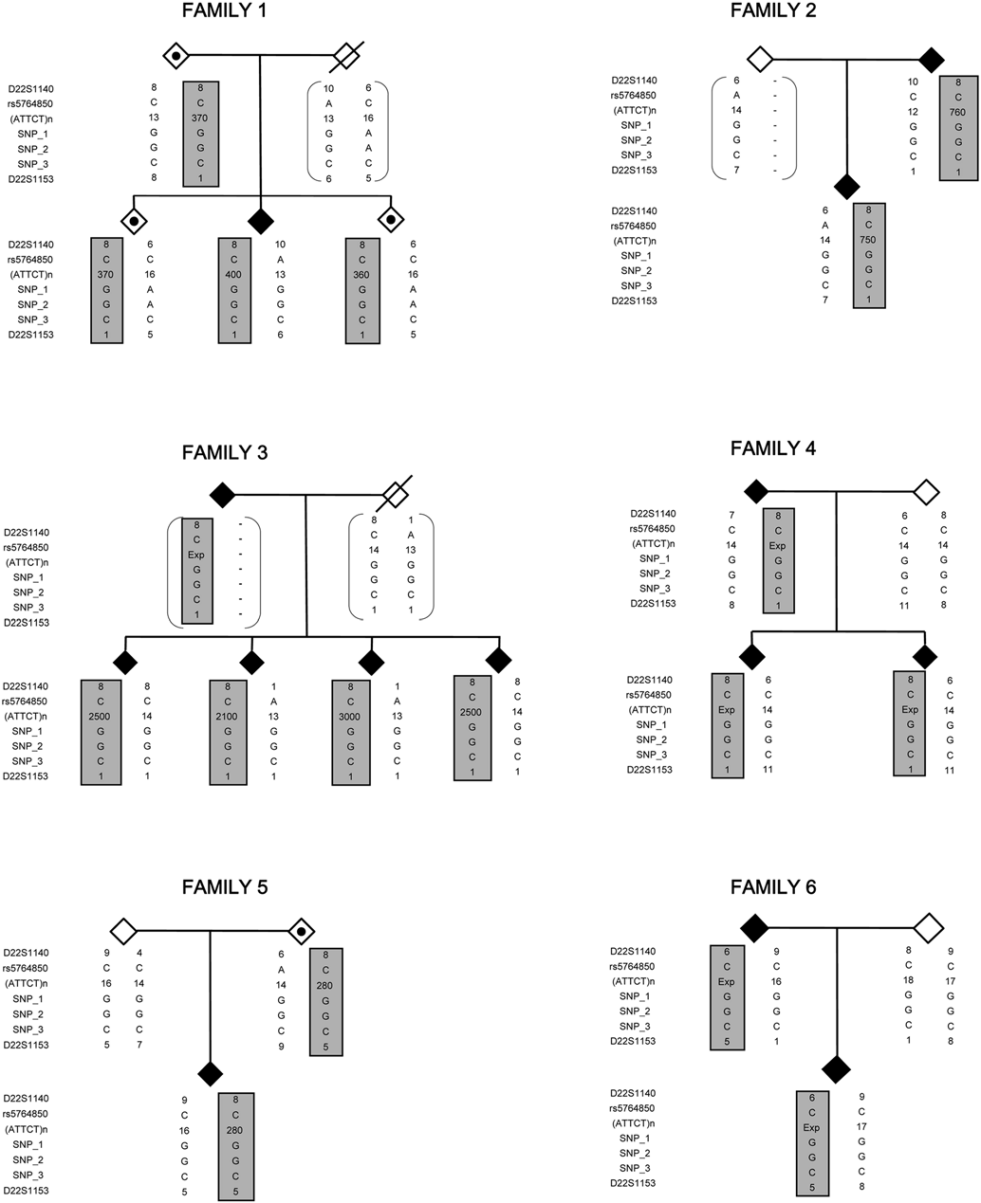
Pedigrees and Haplotypes of Brazilian and Mexican Families. Symbols with black dots indicate asymptomatic carriers of expanded alleles. Haplotypes of four SNPs and 2 STRs, spanning a 1.7 kb region, flanking the repeat are shown. Haplotypes that segregate with the expansion are boxed.

Haplotype analysis with the informative SNPs and the STRs showed that all expanded chromosomes in the Brazilian families shared the 8CGGC1 haplotype, also observed in the large Mexican family 4 (Figure 1). In the Mexican family 6, the 6CGGC5 haplotype was transmitted with the expansion, whereas the mother-child pair (with a 280 repeat allele) from family 5 had the 8CGGC5 haplotype. The results (Table 1) showed that the CGGC intragenic haplotype was conserved among all the SCA10 families, and was associated with three surrounding haplotypes with low frequencies in control subjects. LD analysis showed significant results for haplotype 8CGGC1, overall and in Brazilians. Haplotype 8CGGC5 varied from the former at marker D22S1153, possibly resulting from it by recombination. The additional haplotype, 6CGGC5, seems to have been originated from the latter by one more step of recombination, at marker D22S1140.

**Table 1:** Overall and by Population LD Analysis.

Haplotype	Frequency		δ	P-value
	Control Subjects	Subjects with SCA10		
*Overall*				
CGGC	.70	1	1	NS
CAAC	.03	0	…	…
AAAC	.08	0	…	…
AGGC	.18	0	…	…
AGGT	.01	0	…	…
8CGGC1	.16	.67	.61	.011
6CGGC5	.03	.16	.14	NS
8CGGC5	.01	.16	.15	NS
*Brazilian*				
CGGC	.71	1	1	NS
CAAC	.02	0	…	…
AAAC	.08	0	…	…
AGGC	.19	0	…	…
AGGT	0	0	…	…
8CGGC1	.12	1	1	.000
6CGGC5	.04	0	…	…
8CGGC5	0	0	…	…
*Mexican*				
CGGC	.73	1	1	NS
CAAC	.03	0	…	…
AAAC	.06	0	…	…
AGGC	.18	0	…	…
AGGT	0	0	…	…
8CGGC1	.21	.33	.15	NS
6CGGC5	.01	.33	.32	NS
8CGGC5	.03	.33	.30	NS

NS = not significant.

**Figure 2:**
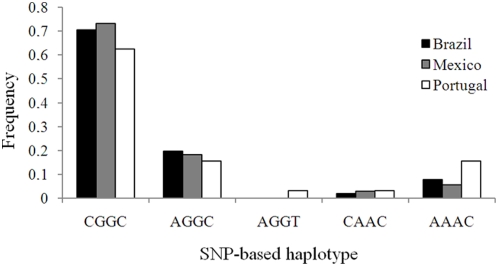
Frequency of SNP-based Haplotypes. Frequency of SNP-based, intragenic, haplotypes of *ATXN10*, in Mexican, Brazilian and Portuguese control populations.

Among the 154 control chromosomes of Brazilian (51), Mexican (71) and Portuguese (32) origin (Figure 2 and Table 1) only five of the 16 possible combinations of the four SNPs were observed. The three control populations had CGGC as the most common haplotype (70%), followed by AGGC (18%). Globally, AAAC was rare, except in Portugal, where it attained a frequency similar to that of AGGC. The two other haplotypes, AGGT and CAAC, were even rarer, AGGT being observed only in the Portuguese controls.

Analysis of the intragenic together with repeat flanking polymorphisms showed 33 haplotypes among Brazilian control chromosomes (Table S1); from those associated also with the SCA10 expansion, 8CGGC1 was the most common (11.8%), 6CGGC5 had a frequency of 3.9%, and 8CGGC5 was absent; the other haplotypes ranged 7.8–2.0%. The Mexican control population was even more diverse, showing 43 different haplotypes (Table S1); disease-associated haplotypes were found to be more frequent, with 8CGGC1 accounting for 21.1%, followed by 8CGGC5 (2.8%) and 6CGGC5 (1.4%); additional haplotypes varied from 5.6 to 1.4%. The Portuguese controls showed 25 haplotypes (Table S1); the 8CGGC1 (12.5%) and 6CGGC5 (3.1%) were the only disease-shared found; the others ranged 6.3–3.1%.

**Figure 3:**
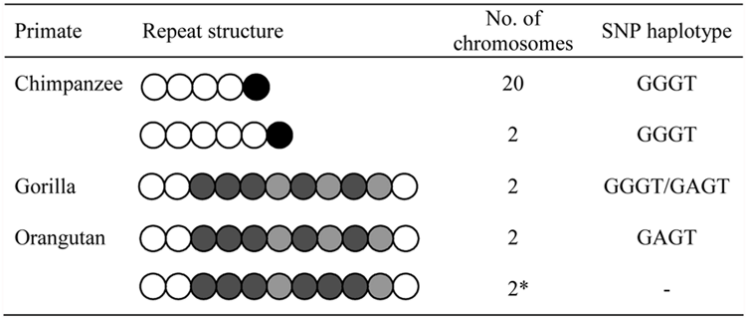
Repeat Structure and Haplotype in non-Human Primates. Open circles represent ATTCT repeats; black circles represent ATTTAT repeats; light gray circles represent GTTCT repeats and dark gray circles represent GTTCC repeats; * Repeat structure from the Orangutan database.

To investigate repeat evolution and ancestral SNP status at the *ATXN10* locus, we assessed repeat tract configuration and SNP genotypes in non-human primates. Repeat structures for the 11 chimpanzees, two gorillas and one orangutan studied are shown in Figure 3. Concerning the SNP background, an ancestral GAGT haplotype was observed both in gorilla and orangutan. A derived haplotype, only one step apart from the ancestral (GGGT), was observed in all chimpanzees and one out of two gorillas examined. None of these haplotypes were observed in humans.

**Figure 4:**
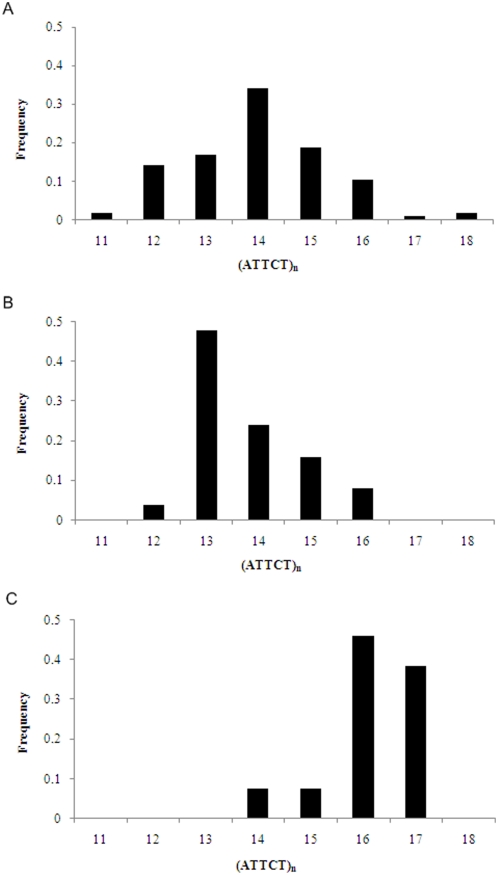
Distribution of Normal Alleles According to SNP Lineages. Repeat distribution of normal alleles in the three most common lineages A. CGGC (n = 105), B. AGGC (n = 25), and C. AAAC (n = 13).

Next, to gain some insight into the evolution of normal human *ATXN10* alleles, we analyzed (ATTCT)_n_ allele distribution according to the SNP lineages (Figure 4). The most ancestral CGGC showed a modal distribution, with (ATTCT)_14_ being the modal allele. The derived AGGC may have arisen on a background carrying the allele with 13 repeats, from which variation may have been generated, most commonly by the addition of one repeat until reaching (ATTCT)_16_. The AAAC lineage was less frequent, with alleles with 16 and 17 repeats overrepresented, when compared to alleles with 14 and 15 repeat units. We next studied the expansion process and compared genetic distance (measured by the variation accumulated on flanking STRs) among different-size alleles carrying the CGGC lineage. The flanking haplotypes of alleles 12–16 repeats showed similarity by pairwise comparison, with R_ST_ as the distance calculation method, by using Arlequin software. This result indicates that little genetic distance exists among these *ATXN10* alleles (Table S2).

## Discussion

The finding that SCA10 families originating from Latin America shared the same intragenic haplotype strongly suggests their common ancestry. The sharing of the same or closely related STR-haplotypes, by families of both origins, lead us to propose a single SCA10 mutational event in these populations. The 6CGGC5 haplotype seems to have been derived from 8CGGC5, which in turn originated from 8CGGC1, the most common and probably the ancestral haplotype. The higher frequency of this haplotype in the Mexican control population, and the greater diversity at the STR markers in Mexican SCA10 families suggest a Mexican Amerindian origin for the SCA10 expansion. The population of Central America is genetically poorly studied as concerns these neurodegenerative diseases; thus, there is a gap between Mexico and Brazil, regarding the spread of SCA10 mutation along the Continent. Brazil and Argentina are traditionally related through the Gaucho, inhabitants of the Pampa region. This region includes parts of Argentina, Uruguay and southern Brazil. The Gaucho community was originated from the admixture of Amerindians, European colonizers and Africans [11]. The Amerindian North-South migrations might explain the spread of the SCA10 expansion from Mexico to these populations.

Our results indicate that alleles of intermediate size, in SCA10, are originated from contraction of fully expanded alleles and, thus, they do not represent a premutation stage. This suggests that new arisen mutations are very unlikely in SCA10, which is supported by the restricted geographic origin of SCA10 families.

Human normal alleles with 11 to 16 repeats have previously been shown to contain pure ATTCT tracts [12]. Contrary to the normal repeat length in humans, all the non-human primates revealed a smaller number of repeat motifs (11 or fewer), indicating that the increase in repeat size occurred after the *Homo-Pan* split about 6.3 million years ago [13]. Moreover, in all primates studied the ATTCT motif is followed by an interrupted ATTCT-like sequence. A similar pattern has also been observed in 71% of human normal alleles over 17 repeats, comprising interruptions of ATTGT followed by TTTCT or only TTTCT [12].

The molecular mechanisms of repeat instability, regarding contractions and expansions, are mediated by DNA replication, repair and recombination, probably concerted [14]. To test the replication slippage model, we analyzed normal allele distributions in each SNP lineage and compared genetic distance among different-size alleles, measured by the diversity on flanking STRs. Alleles of different size, from the same lineage, showed little or null genetic distance. This indicates that normal alleles up to 18 ATTCT repeats originated by a single step mechanism of repeat length mutation.

The ATTCT repeat is highly unstable both in terms of repeat length (range from 280–4500 repeats) and structure. Alleles of intermediate size, in family 5, have revealed multiple repetitive ATGCT repeats in the most proximal part of the expansion and ATTCTAT septanucleotide repeats in its distal part, whereas larger alleles, in family 4, have showed two different septarepeat interruptions ATTTTCT and ATATTCT [12]. The Brazilian family 3 and the Mexican family 4, that share the 8CGGC1 haplotype, as well as the Mexican family 5, with the 8C280GGC5 haplotype, have interruptions in their ATTCT repeat tract. Notwithstanding, the constancy revealed by the flanking SNP haplotype and the repeat interruptions, suggests the involvement of gene conversion events in the generation of expanded alleles [15]. This hypothesis is also favored by the genomic context in which the repeat itself is located. Repeat Masker computation [16] of intron 9 of the *ATXN10* revealed repetitive elements, including 23 Alu and 29 LINE repeat sequences, representing 32% of the total intron length. These elements are known to promote microsatellite mutability through gene conversion [17]. The small number of alleles observed in non-human primates seems to be illustrative of a recent introgression of these sequences into the human genome.

In conclusion, we show evidence of an ancestral common origin for SCA10 in Latin American populations, which might have arisen in the Amerindian population and later, have been spread into the mixed populations of Mexico and Brazil.

## Materials and Methods

### Subjects

Six unrelated families with SCA10 were studied; three were Brazilian families of mixed Portuguese and Amerindian ancestry from Rio Grande do Sul; two were the Mexican families used to map the *ATXN10* gene [1]–[2]; and the last one was an early-onset Mexican patient with an unusual allele of 280 repeats inherited from his asymptomatic mother [12]. Two of the Brazilian families (Figure 1: Families 1 and 2) were described as suffering from pure ataxia [5], while the third was newly identified and presented ataxia and seizures. A total of 34 carriers of the SCA10 expansion and 20 relatives were analyzed. Forty-four families from the normal population (20 Brazilians, 10 Portuguese and 14 Mexicans) were also studied. Peripheral blood samples were collected after written informed consent. Genomic DNA was obtained from peripheral blood leucocytes by standard techniques [18].

### Methods

Repeat sizes at the *ATXN10* gene were assessed by PCR amplification with flanking primers and Southern blot as reported elsewhere [5]. Amplification of the polymorphic regions was performed with primer sequences listed in Table 2; each PCR reaction was carried out with 1 µM of each primer, 200 µM deoxynucleotides, 1.5 mM MgCl_2_, 1 U of Taq polymerase and 2% of formamide in a final volume of 25 µL. SNPs were detected by dHPLC (Transgenomic, Omaha, NE) and further identified by sequencing. STR allele sizes were analyzed in a 310 ABI PRISM genetic analyzer (Applied Biosystems, Foster City, CA) and using GenScan software (Applied Biosystems, Foster City, CA). SNPs were selected from the dbSNP database (NCBI) or identified during this work (Table 2). Haplotypes were reconstructed with informative SNPs and STRs D22S1140 and D22S1153, spanning a region of ∼1.7 Mb, flanking the repeat on both sides.

**Table 2:** SNPs Selected for the Haplotype Study.

SNP	RefSNP ID	Base substitution	Distance from the (ATTCT)_n_ (bp)	Primer sequence (5′- 3′)	Annealing conditions
A	rs136002	A>G^a^	−2045	F-TGTTGATGTCCAACAGACTTTTC	56.4°, 30 sec
				R-GAATTATTTGTTAAAGAGAATCGTGAA	
B	rs5765626	G>A^a^	−1957	The same as for SNP A	56.4°, 30 sec
C	rs5764850	A>C^a^	−1198	F-TCTAGGAAAAGGGTGGCAACT	58°, 30 sec
				R-CATGTCCTGGAGAGGTAGG	
D	rs136003	−/A^a^	−898	F-TCTAGGAAAAGGGTGGCAACT	58°, 30 sec
				R-AGCCAAAACCAAAAGCTGGT	
E	SNP_1	G>A^b^	+47	F-TTGAGATGAAGTCTCTCTATGTTGC	57.5°, 30 sec
				R-AAAGACAAGACAGGCATAGGAAA	
F	SNP_2	G>A^b^	+303	The same as for SNP E	57.5°, 30 sec
G	SNP_3	C>T^b^	+370	The same as for SNP E	57.5°, 30 sec
H	rs136005	C>T^a^	+1091	F-CTCCCCTTTTGAAACCCCTA	56.4°, 30 sec
				R-GAGTCTTGCCTTTCAAAATCCA	
I	rs9614518	A>T^a^	+1296	The same as for SNP H	56.4°, 30 sec
J	rs6006808	A>G^a^	+1338	The same as for SNP H	56.4°, 30 sec
K	rs11912672	A>G^a^	+1576	F-TGGATTTTGAAAGGCAAGACTC	56.4°, 30 sec
				R-AAGAACGCTGATATCTCCGATTATG	
L	rs9614781	C>G^a^	+1638	The same as for SNP K	56.4°, 30 sec

a According to NCBI, HapMap and Genome Browser database.

b New SNPs found in this study and submitted to the NCBI.

Differences in the overall distribution of alleles on normal and disease chromosomes were tested by Fisher's exact test. Evidence for LD was established using δ = (F_d_−F_c_)/(1−F_c_), where F_d_ is the frequency of carrier and *F*_c_ is the frequency of noncarrier chromosomes [19]. Non-segregating haplotypes in SCA10 families were used to determine haplotypes frequency.

Genetic distances among different-size normal alleles were calculated assuming a stepwise mutation model for D22S1140 and D22S1153 using R_ST_. R_ST_ is an analogue of F_ST_ that takes into account differences in repeat units from STR alleles when estimating genetic distances among STR haplotypes. Analyses were performed in Arlequin ver 3.11 [20].

## Supporting Information

Table S1 Haplotype frequencies by control population
(0.05 MB DOC)

Table S2 Haplotype Frequencies in Controls by Lineage and ATXN10 allele
(0.23 MB DOC)
