# Supplementary material for: Ancestral Origin of the ATTCT Repeat Expansion in Spinocerebellar Ataxia Type 10 (SCA10)
Source: PLoS One. 2009 Feb 23;4(2):e4553. doi: 10.1371/journal.pone.0004553 (PMC2639644; doi:10.1371/journal.pone.0004553)
Supplement: Table S1 — Haplotype frequencies by control population (0.05 MB DOC) [file pone.0004553.s001.doc]

Table S1. Haplotype frequencies by control population

| Brazilian (n=51) | | Mexican (n=71) | | Portuguese (n=32) | |
| --- | --- | --- | --- | --- | --- |
| Haplotype | Frequency (%) | Haplotype | Frequency (%) | Haplotype | Frequency (%) |
| 1 AAAC 7  1 AGGC 1  1 CGGC 1  1 CGGC 4  1 CGGC 5  4 CGGC 1  4 CGGC 7  5 AGGC 1  5 AGGC 2  5 CGGC 2  6 AAAC 7  6 AGGC 1  6 AGGC 2  6 AGGC 7  6 CAAC 5  6 CGGC 1  6 CGGC 2  6 CGGC 4  6 CGGC 5  7 AAAC 5  7 AGGC 1  7 CGGC 1  8 AGGC 1  8 CGGC 1  8 CGGC 8  8 CGGC 4  9 CGGC 5  10 AGGC 6  10 AAAC 1  10 CGGC 1  10 CGGC 5  10 CGGC 8  11 CGGC 1 | 2.0  2.0  2.0  3.9  5.9  2.0  2.0  2.0  2.0  2.0  2.0  3.9  2.0  2.0  2.0  3.9  3.9  2.0  3.9  2.0  2.0  2.0  2.0  11.8  2.0  3.9  2.0  2.0  2.0  7.8  5.9  2.0  2.0 | 1 AAAC 9  1 AGGC 2  1 CAAC 1  1 CGGC 1  1 CGGC 5  1 CGGC 8  4 CGGC 5  4 CGGC 7  5 CAAC 7  5 CGGC 7  6 AGGC 4  6 AGGC 5  6 AGGC 9  6 CGGC 1  6 CGGC 11  6 CGGC 5  6 CGGC 8  7 AGGC 1  7 AGGC 6  7 CGGC 1  7 CGGC 4  7 CGGC 5  7 CGGC 8  8 AAAC 2  8 AAAC 5  8 AGGC 1  8 AGGC 2  8 AGGC 4  8 AGGC 7  8 AGGC 9  8 CGGC 1  8 CGGC 10  8 CGGC 5  8 CGGC 6  8 CGGC 7  8 CGGC 8  9 CGGC 1  9 CGGC 5  9 CGGC 8  10 AAAC 6  10 AGGC 7  10 CGGC 4  11 CGGC 5 | 1.4  1.4  1.4  2.8  2.8  1.4  1.4  1.4  1.4  1.4  1.4  1.4  1.4  2.8  1.4  1.4  1.4  1.4  1.4  2.8  1.4  2.8  1.4  1.4  1.4  1.4  2.8  1.4  1.4  1.4  21.1  1.4  2.8  1.4  5.6  2.8  4.2  1.4  1.4  1.4  1.4  1.4  2.8 | 1 AGGC 1  1 CGGC 1  2 CAAC 5  4 AGGC 6  4 AGGT 5  4 CGGC 4  5 AGGC 1  5 CGGC 4  6 AAAC 5  6 AAAC 7  6 AGGC 7  6 CGGC 5  6 CGGC 7  7 CGGC 1  8 AAAC 5  8 CGGC 1  8 CGGC 4  8 CGGC 8  9 AAAC 1  9 AGGC 7  9 CGGC 1  9 CGGC 4  10 CGGC 1  10 CGGC 5  10 CGGC 7 | 3.1  6.3  3.1  3.1  3.1  3.1  3.1  3.1  3.1  6.3  3.1  3.1  3.1  3.1  3.1  12.5  6.3  3.1  3.1  3.1  3.1  6.3  3.1  3.1  3.1 |
