# Supplementary material for: Ancestral Origin of the ATTCT Repeat Expansion in Spinocerebellar Ataxia Type 10 (SCA10)
Source: PLoS One. 2009 Feb 23;4(2):e4553. doi: 10.1371/journal.pone.0004553 (PMC2639644; doi:10.1371/journal.pone.0004553)
Supplement: Table S2 — Haplotype Frequencies in Controls by Lineage and ATXN10 allele (0.23 MB DOC) [file pone.0004553.s002.doc]

Table S2. Haplotype Frequencies in Controls by Lineage and *ATXN10* allele

|  |  |  | Frequency (%) | | |
| --- | --- | --- | --- | --- | --- |
| Lineage | *ATXN10* allele size | STR haplotype | Brazilian (n=51) | Mexican (n=71) | Portuguese (n=32) |
| AGGC | 12 | 8-2 |  | 1.41 |  |
|  | 13 | 1-1 | 1.96 |  | 3.12 |
|  |  | 1-2 |  | 1.41 |  |
|  |  | 4-6 |  |  | 3.12 |
|  |  | 5-1 |  |  | 3.12 |
|  |  | 5-2 | 1.96 |  |  |
|  |  | 6-1 | 1.96 |  |  |
|  |  | 6-2 | 1.96 |  |  |
|  |  | 7-1 | 1.96 | 1.41 |  |
|  |  | 8-1 |  | 1.41 |  |
|  |  | 8-7 |  | 1.41 |  |
|  |  | 10-6 | 1.96 |  |  |
|  |  | 10-7 |  | 1.41 |  |
|  | 14 | 6-1 | 1.96 |  |  |
|  |  | 6-5 |  | 1.41 |  |
|  |  | 6-7 | 1.96 |  | 3.12 |
|  |  | 6-9 |  | 1.41 |  |
|  |  | 8-1 | 1.96 |  |  |
|  |  | 9-7 |  |  | 3.12 |
|  | 15 | 5-1 | 1.96 |  |  |
|  |  | 7-6 |  | 1.41 |  |
|  |  | 8-2 |  | 1.41 |  |
|  |  | 8-9 |  | 1.41 |  |
|  | 16 | 6-4 |  | 1.41 |  |
|  |  | 8-4 |  | 1.41 |  |
| AAAC | 14 | 6-7 |  |  | 3.12 |
|  | 15 | 6-7 |  |  | 3.12 |
|  | 16 | 1-7 | 1.96 |  |  |
|  |  | 1-9 |  | 1.41 |  |
|  |  | 6-7 | 1.96 |  |  |
|  |  | 7-5 | 1.96 |  |  |
|  |  | 9-1 |  |  | 3.12 |
|  |  | 10-1 | 1.96 |  |  |
|  | 17 | 6-5 |  |  | 3.12 |
|  |  | 8-2 |  | 1.41 |  |
|  |  | 8-5 |  | 1.41 | 3.12 |
|  |  | 10-6 |  | 1.41 |  |
| CGGC | 11 | 4-4 |  |  | 3.12 |
|  |  | 10-5 | 1.96 |  |  |
|  | 12 | 1-4 | 1.96 |  |  |
|  |  | 1-5 | 1.96 |  |  |
|  |  | 6-2 | 1.96 |  |  |
|  |  | 7-5 |  | 1.41 |  |
|  |  | 7-8 |  | 1.41 |  |
|  |  | 8-1 |  | 2.82 |  |
|  |  | 8-4 | 3.92 |  | 6.24 |
|  |  | 9-4 |  |  | 3.12 |
|  |  | 10-1 | 3.92 |  | 3.12 |
|  |  | 11-1 | 1.96 |  |  |
|  | 13 | 1-4 | 1.96 |  |  |
|  |  | 1-5 | 1.96 |  |  |
|  |  | 4-1 | 1.96 |  |  |
|  |  | 5-2 | 1.96 |  |  |
|  |  | 6-4 | 1.96 |  |  |
|  |  | 6-5 |  | 1.41 | 3.12 |
|  |  | 7-5 |  | 1.41 |  |
|  |  | 8-1 | 1.96 | 4.22 |  |
|  |  | 8-5 |  | 1.41 |  |
|  |  | 8-8 | 1.96 |  | 3.12 |
| CGGC |  | 9-4 |  |  | 3.12 |
|  |  | 9-5 | 1.96 |  |  |
|  |  | 10-1 | 1.96 |  |  |
|  |  | 10-5 | 1.96 |  |  |
|  | 14 | 1-1 |  |  | 3.12 |
|  |  | 1-5 | 1.96 | 1.41 |  |
|  |  | 4-7 |  | 1.41 |  |
|  |  | 6-1 | 1.96 | 1.41 |  |
|  |  | 6-2 | 1.96 |  |  |
|  |  | 6-5 | 3.92 |  |  |
|  |  | 6-7 |  |  | 3.12 |
|  |  | 6-8 |  | 1.41 |  |
|  |  | 6-11 |  | 1.41 |  |
|  |  | 7-1 | 1.96 |  | 3.12 |
|  |  | 8-1 | 7.84 | 8.45 | 12.5 |
|  |  | 8-6 |  | 1.41 |  |
|  |  | 8-7 |  | 2.82 |  |
|  |  | 8-8 |  | 1.41 |  |
|  |  | 9-1 |  |  | 3.12 |
|  |  | 10-1 | 1.96 |  |  |
|  |  | 10-4 |  | 1.41 |  |
|  |  | 10-5 | 1.96 |  | 3.12 |
|  | 15 | 1-1 | 1.96 | 1.41 | 3.12 |
|  |  | 1-5 |  | 1.41 |  |
|  |  | 1-8 |  | 1.41 |  |
|  |  | 4-5 |  | 1.41 |  |
|  |  | 4-7 | 1.96 |  |  |
|  |  | 5-7 |  | 1.41 |  |
|  |  | 6-1 | 1.96 |  |  |
|  |  | 7-1 |  | 1.41 |  |
|  |  | 7-4 |  | 1.41 |  |
|  |  | 8-1 |  | 4.22 |  |
|  |  | 8-7 |  | 2.82 |  |
|  |  | 8-8 |  | 1.41 |  |
|  |  | 10-8 | 1.96 |  |  |
|  |  | 11-5 |  | 2.82 |  |
|  | 16 | 1-1 |  | 1.41 |  |
|  |  | 5-4 |  |  | 3.12 |
|  |  | 6-1 |  | 1.41 |  |
|  |  | 7-1 |  | 1.41 |  |
|  |  | 8-1 | 1.96 |  |  |
|  |  | 8-10 |  | 1.41 |  |
|  |  | 9-1 |  | 4.22 |  |
|  |  | 9-5 |  | 1.41 |  |
|  |  | 10-7 |  |  | 3.12 |
|  | 17 | 9-8 |  | 1.41 |  |
|  | 18 | 8-1 |  | 1.41 |  |
|  |  | 8-5 |  | 1.41 |  |
| AGGT | 14 | 4-5 |  |  | 3.12 |
| CAAC | 12 | 5-7 |  | 1.41 |  |
|  | 16 | 2-5 |  |  | 3.12 |
|  |  | 6-5 | 1.96 |  |  |
|  | 18 | 1-1 |  | 1.41 |  |
